# Entanglement of Genetics and Epigenetics in Parkinson’s Disease

**DOI:** 10.3389/fnins.2019.00277

**Published:** 2019-03-29

**Authors:** H. J. van Heesbeen, Marten P. Smidt

**Affiliations:** Faculty of Science, Swammerdam Institute for Life Sciences, University of Amsterdam, Amsterdam, Netherlands

**Keywords:** Parkinson, brain, development, neurdegeneration, genetics, epigenetics

## Abstract

Parkinson disease (PD) is a common neurodegenerative disorder that progresses with age, with an increasing number of symptoms. Some of the efforts to understand PD progression have been focusing on the regulation of epigenetic mechanisms, that generally include small molecular modifications to the DNA and histones that are essential for regulating gene activity. Here, we have pointed out difficulties to untangle genetic and epigenetic mechanisms, and reviewed several studies that have aimed for untangling. Some of those have enabled more solid claims on independent roles for epigenetic mechanisms. Hereby, evidence that specific DNA hydroxymethylation, global hyperacetylation, and histone deacetylase (HDAC) dependent regulation of *SNCA*, one of the hallmark genes involved in PD, have become more prominent from the current perspective, than mechanisms that directly involve DNA methylation. In the absence of current epigenetic clinical targets to counteract PD progression, we also hypothesize how several mechanisms may affect local and global epigenetics in PD neurons, including inflammation, oxidative stress, autophagy and DNA repair mechanisms which may lead to future therapeutic targets.

## Introduction

Parkinson disease (PD) is a common neurodegenerative disorder, with a variety of motor and non-motor symptoms (Poewe et al., 2017). The progressive decline of fluid movements in PD patients results from reduced dopamine (DA) production in the Substantia Nigra compacta (SNc) and especially a lack of delivery to the dorsal striatum. Since these symptoms have been described by James Parkinson in 1817, an impressive progress has been made in understanding the underlying pathology. For this purpose, the neural circuits, pathological hallmarks and the affected anatomical regions have been extensively characterized (Parkinson, 2002; Przedborski, 2017). At this exciting time, the mechanisms that could determine the onset and drive PD progression, are getting in focus (Przedborski, 2017).

The low prevalence of familial PD suggests that stochastic events, for instance environmental cues, pathogens and lifetime somatic mutations of both nuclear and mitochondrial DNA may all contribute to PD. These ideas have been used as well to explain the relatively low concordance of PD that mono-zygotic twins show while they sprout from a single cell (Tanner et al., 1999). Next to this, accumulating studies have found correlations between PD and lifestyles, that in some aspects may be causal to differences in PD concordance, like the possible protective effect of smoking (Hernán et al., 2002; Derkinderen et al., 2014). However, extracellular cues, protein homeostasis, genetics, and epigenetics are extensively entangled. In the current era, new technologies have put neuroscience on the branch of untangling these components, increasingly enabling more solid claims on protective lifestyles or therapies for PD. Hence, here we review how far studies have gone forward in untangling genetics and epigenetics and hypothesize how various epigenetic mechanisms may facilitate PD progression.

Since PD shares some characteristics with aging, like DNA and mitochondrial damage, together with an increase in (neuro)inflammation (Ransohoff, 2016; Poewe et al., 2017; Surmeier et al., 2017) as well as an increase of prevalence with age (Pringsheim et al., 2014), we also consider how such processes may influence the epigenome in PD to summarize potential therapeutic epigenetic targets to slow down PD progression.

## The Definition of Epigenetic

The term epigenetic has been established to appoint the intriguing phenomenon that equal genomes give rise to different gene activity states (Deans and Maggert, 2015). The term epigenetic seems to be heavily exposed to semantic drift, by which the meaning of a word changes over time. A fueling factor hereof seems the increasing knowledge on the mechanisms that are involved in regulating different activity states, but that were largely unknown at the time the term was originally postulated to capture a certain feature of biological development (Deans and Maggert, 2015). Considering neuroscience, semantic drift of epigenetics has been driven by the simple use of the term in the field, which influenced the debate if meiotic/mitotic inheritance should be part the definition, since such a limitation would simply mean that the term could not be applied to post-mitotic neurons that do not divide. Still, the term is used effectively and very broadly in the field. We believe that nowadays the majority of researchers will primarily think of the modifications to DNA and histones or chromatin when they are asked to imagine epigenetics. In line with this, many have suggested and followed suggestions to limit epigenetics to the chromosomal level (Bird, 2007; Benayoun et al., 2015; Deans and Maggert, 2015; Allis and Jenuwein, 2016). This more literal (*epi* translates ‘near’) use of the term seems to increase the usefulness of the term also for pragmatic reasons. As is exemplified by one of the most debatable members of the epigenetic family, micro-RNAs. When compared to the plethora of chemical DNA and histone modifications, micro-RNAs have a very clarifying and distinctive name that does not necessarily benefit being over-arched by a term as epigenetics. In line with this, we consider the epigenome as the pattern of chemical modifications to histone proteins and DNA.

Finally, changes in epigenetic marks may not always change expression states directly, or lead to measurable expression fluctuations (e.g., equal transcript levels), still, such changes we will call epigenetic changes. Therefore, we find that the broadly used definition of epigenetics by Adrian Bird complies these criteria best: “the structural adaptation of chromosomal regions so as to register, signal or perpetuate altered activity states” (Bird, 2007). To us, this includes histone proteins and their modifications, DNA modifications and non-coding RNAs that are structural to chromatin.

## Hallmarks of PD

Familial PD associates with mutations in genes that are often involved in Ca^2+^ regulation, mitochondrial oxidation pathways and proteosomal/autophagy overload or capacity (Klein and Westenberger, 2012). In line with this, sporadic PD seems most harmful for a selective group of neurons especially in the SNc—but also outside the midbrain—that have specific morphological and energetic characteristics, like broad axonal branching with a large number of mitochondria and atypical calcium regulation wherein accumulated protein aggregates are found (Surmeier et al., 2017).

Next to neuronal loss, α-Synuclein (α-Syn)-positive cellular inclusions called Lewy bodies (LBs) are the most prominent post-mortem pathological hallmark though perhaps not essential for clinical PD (e.g., symptoms) or neuronal degeneration (Engelender and Isacson, 2017; Surmeier et al., 2017). Moreover, depending on the (mosaic) genetic background of each individual PD patient, it is unclear if α-Syn accumulation act as an initiator of PD, a driving factor of symptomatic deterioration or a mere final burden in already diseased neurons (Engelender and Isacson, 2017; Surmeier et al., 2017).

Current debates on the progression of PD mainly focus on the apparent spreading of the disease from vulnerable to less vulnerable neuronal subtypes and regions, but whether this just reflects the divergent vulnerability of neuronal subsets and whether spreading is facilitated by α-Syn subforms in a prion-like fashion or, for instance through local inflammatory processes, is still under debate (Engelender and Isacson, 2017). On top of this, the sheer over-expression of α-Syn as seen in patients with duplications of the *SNCA* gene, show largely divergent phenotypes, with the symptomatic onset varying from 18 to 77 years of age in one report, or even lacking full penetrance in another. Even when the *SNCA* gene is triplicated, it may take decades before the first symptoms are observed and decades more for the disease to become lethal (Nishioka et al., 2006; Byers et al., 2011; Konno et al., 2016). From a different perspective, newly transplanted grafts can survive over 20 years within a progressed PD brain and without broad LB-formation (Li et al., 2008; Mendez et al., 2008). Finally, the long duration of many forms of juvenile PD (Schrag et al., 1998) suggests that aging related mechanisms that are observed in normal aging and other common hallmarks of degenerative disease combined determine the speed of PD progression at different stages and it is tempting to believe that a minor slowdown of PD progression could result, in many cases, in years of higher quality of life.

## Somatic Mutations

**FIGURE 1:**
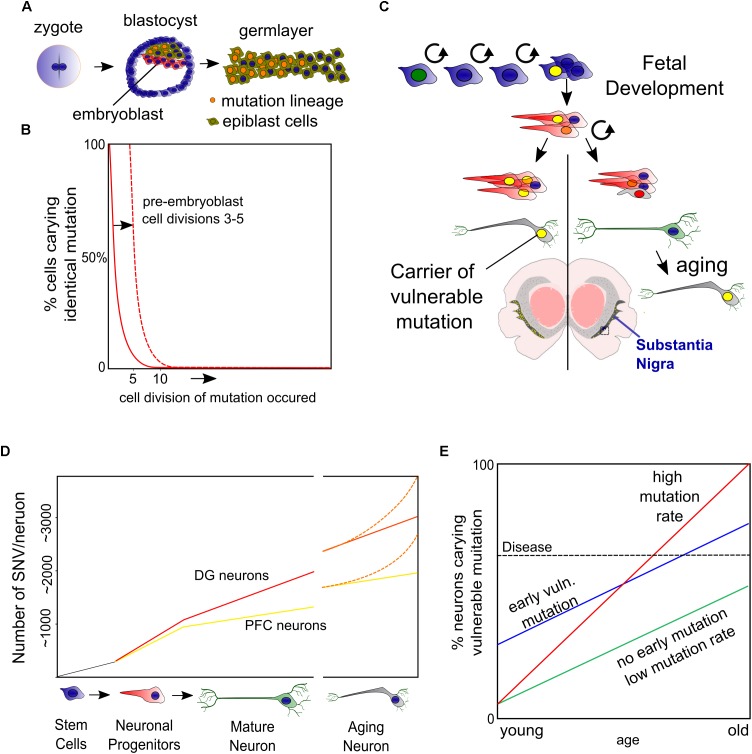
Somatic mutations. **(A)** During the first cell divisions of a zygote the blastocyst is formed of which only a subset (the epiblast) will form the embryo. **(B)** Based on the work of Morris et al. (2010) on blastocyst development, we have depicted a mutation occurring in the 5^*th*^ zygotic cell division and may cause up to ∼25% of the epiblast cells carrying a mutation. Mutations in the early embryonic cell divisions (Ju et al., 2017) increase the chance that a substantial fraction of the embryonic cells carry this burden. **(C)** Not only early embryonic vulnerable mutations (yellow nuclei), but also accumulation of mutations in neuronal progenitors and during post-mitotic life lead to a mosaic and, at least for a fraction of DA neurons to an increased burden. **(D)** We have depicted the accumulation of single nucleotide variants (SNVs) found in early development and adult and aged neurons of the prefrontal cortex (PFC) and dentate gyrus (DG) based on work from the labs of Christopher Walsh and Flora Vaccarino (Lodato et al., 2015, 2018; Bae et al., 2018). **(E)** When mutation rates are equal, an early embryonic mutation can lead to earlier onset of disease. Another aspect thereby is the speed of mutation, which may be increased by specific neuronal characteristics of DA neurons, like oxidative stress or asymmetric cell divisions during development.

Epidemiological studies have linked the exposure of several substances and familial mutations to PD development. Still, the discordance in PD development between mutation carrying relatives or people that share both lifestyles and genes, like monozygotic twins, remains puzzling. It seems that an extra hit, or even multiple hits may be required to further convey an inherited set of susceptible genes into PD. An interesting but under-explored hypothesis is that somatic mutations are involved. Especially point mutations have become more renowned candidates in recent years following the technological advantage of single cell/neuron whole genome sequencing that can detect unique point mutations in a single neuron. These single nucleotide variants (SNVs) may find their origin during early fetal development, when on average already several 100 mutations give rise to unique genomes for each individual neuron (Figure 1A–D) (Perandones et al., 2015; Bae et al., 2018). At the time human neurons have matured, they contain already ∼1500 lifetime mutations and this number slowly increases with age (Figure 1D).

Although the authors show in these elegant studies that the DNA repair related neurodegenerative diseases Cockayne syndrome and Xeroderma Pigmentosum have increased DNA mutation rates (Lodato et al., 2015, 2018), it is unknown if later in life or progressed PD comparable amounts of mutations are found, or perhaps that mutation rates increase as a feature of accelerated aging or even in healthy DA neurons (Figure 1D,E). Nevertheless, it is likely that increasing DNA mutations are an increasing burden of aging DA neurons that sooner or later occur at vulnerable sites, disrupting vital genes or regulatory DNA elements in at least a fraction of neurons (Figure 1E).

If somatic mutations would play a role in the low PD concordance observed in monozygotic twins, somatic mutations in zygotic cells seem to be better candidates than progenitors or matured neurons. Especially when these mutations occur randomly in all genes, which they seem to do, though with a preference for exons (Lodato et al., 2018). Therefore, ‘harmful mutations’ would only occur randomly in a small fraction of the total number of progenitors or neurons. Instead, early embryonic mutations could cause changes in disease onset even when mutation rates remain equal (Figure 1E).

Another interesting aspect herein is that PD progression regularly has a preference to develop earlier and faster on one side of the left-right axis but is most severe when progressing at both sides (Marinus and van Hilten, 2015). To which extend such ‘early’ mutations can contribute to PD development is largely unknown and depends on the mutation rate but also the amount of cell divisions that cells undergo before the DA lineage is split-off (Figure 1). However, there is evidence suggesting that somatic mutations cause unilateral brain diseases like hemimegalencephaly (Poduri et al., 2012). A better knowledge on mutation rates in DA neurons (and others affected in PD) and their development would be most helpful. For instance, we don’t know if DA neurons require more cell divisions, or if they have more cellular stress as progenitors or when maturing compared to others. All could lead to faster accumulating somatic mutations in embryonic stem cells compared to others.

Based on the current knowledge, roughly 6–10 mutations will accumulate upon five zygotic cell divisions (Ju et al., 2017). From the data originating from the Walsh lab (Lodato et al., 2018), roughly 4000 mutations on average will become an unbearable burdens to neuronal populations. Assumable, because at least one affected gene leads to cellular dysfunction. This would roughly mean that 0.1–0.5% of all embryo’s would carry a vulnerable mutation in a substantial number (>30%) of all offspring cells. Although these estimations are rough and theoretical, to us it emphasizes the need to better understand how mutations accumulate specifically in early development and vulnerable neurons like DA neurons of the SNc and if left-right differences in SNVs occur in the SNc.

Finally, when combining the burden of somatic mutations with other hypotheses, like the proposal that α-Syn acts in a prion-like fashion to facilitate spreading of PD pathology from one to another neuron (Masuda-Suzukake et al., 2013), one could argue that a mutation in a few neurons may initiate PD and spreads further. However, the penetrance (relation between familial mutation and PD occurrence) of both familial *SNCA* point mutations and duplication is not 100%. Noteworthy, several familial mutations in genes involved in mitochondrial function or autophagy do have a penetrance of 100% (Goedert, 2001; Schulte and Gasser, 2011).

Altogether, to us it seems that a genetic mosaic that rises during early embryonic development rather than later accumulation of neuron specific mutations may have the potential to cause differences in sporadic PD onset. Opposite, accumulation of mutations during later development and in matured neurons may in the end become a final burden for neuronal homeostasis. Furthermore, genetic mosaicism would have the potential to influence the anatomical sites where the first hallmarks of PD are found and is compatible with both Braak’s hypothesis or ones that include an additional environmental or pathogenic cue as well as the opposing theories like the threshold hypothesis (Braak et al., 2003; Engelender and Isacson, 2017). Future single cell sequencing experiments have to reveal how somatic DNA mutations accumulate during early development in relation to midbrain DA neurons and if the mutation rates are indeed leading to mutations in genes that are essential to DA neuronal homeostasis.

Next to SNVs, also newly incorporated transposable elements (TEs) and changes in copy number variants (CNVs) have been found to exist in a mosaic pattern in the brain (Evrony et al., 2012; McConnell et al., 2013). Although several groups of active TEs are waiting to be investigated, these single neuron genomic studies found on average only 0.6 new LINE1 (long interspersed nuclear elements)-TEs per neuron and no more than 13–40% of matured neurons with CNVs. Therefore, from the current perspective SNVs seem the best genetic candidates to be involved as the onset trigger of PD.

Finally, another category of somatic mutations are found in the mitochondrial DNA (mtDNA). Copy numbers of mtDNA increase in SNc neurons of healthy individuals unlike other neuronal types and exceed numbers found in DA neurons of PD patients (Dölle et al., 2016). Although there is some controversy if point mutations accumulate in mtDNA of PD patients, accumulation of mtDNA in healthy SNc has been proposed as a mechanism to maintain a sufficient pool of wild-type mtDNA in healthy individuals (Dölle et al., 2016). Nevertheless, by any calculation the number of mitochondrial genes in nuclear DNA exceeds the couple of dozen genes in mtDNA by far, and there is no clear indication that the observed reduction of mtDNA copy number in PD is the consequence of mtDNA damage but may as well be affected by deregulation of nuclear genes or protein regulation involved in mitochondrial homeostasis.

## Entanglement of Genetics and Epigenetics

In various ways genetics and epigenetics are entangled. Mutations in *cis* and *trans* regulatory DNA elements can affect epigenetics elsewhere, mutations could cause the loss of epigenes (genes that encode epigenetic modifiers) and even undetectable timely changes to the DNA may cause substantial epigenetic defects, like epigenetic modification following DNA repair (Mortusewicz et al., 2005; Oberdoerffer et al., 2008). Vice versa, epigenetic deregulation can cause genomic instability like reactivation of TEs (Benayoun et al., 2015; Pal and Tyler, 2016) which in turn affects the original epigenome. This mechanistic entanglement between the DNA and the epigenome seems vulnerable to form a vicious circle wherein accumulating DNA mutations and deregulation of epigenetic mechanisms could bidirectionally affect each other. However, untangling genetics and epigenetics has been proven more difficult than perhaps initially reasoned. One of the reasons is that epigenetic studies require per definition equal DNA between different experimental conditions. As we have discussed above, DNA mutagenesis may be an ongoing process in virtually all models used. As such, genomes differ already between organs and somatic mutations have accumulated in induced pluripotent stem cells of elderly (Blokzijl et al., 2016; Lo Sardo et al., 2016), which is only addressed properly in some experiments since sequencing the genome of multiple clones or increasing sample numbers are costly processes that, though, decline rapidly at the moment. Here, in some cases, we have accepted epigenetic mechanisms to be independent when they have common features between larger groups of individuals or when the discordance between epigenomic signatures is likely to surmount a plausible amount of *cis*-regulatory DNA mutations. Generally, we have focused on signaling routes that have the potential to globally change epigenome dynamics, since they have the potential to be targets of intervention.

## Untangling Genetics and Epigenetic Drift

In aging individuals, several independent studies have favored the hypothesis that *cis-*regulatory DNA mutations alone unlikely explain (global) epigenetic and transcriptional changes or that they go hand in hand during aging (Fraga et al., 2005; Martin, 2005; Kaminsky et al., 2009; Bui et al., 2015; Jones et al., 2015). In these pioneering studies however, lifetime occurring somatic DNA mutations may have been underexposed as, for instance, mono-zygotic twins have been assumed to contain equal DNA (Kaminsky et al., 2009). In a more recent attempt to unravel genetics and epigenetics, van Baak and colleagues have introduced the term epigenetic *supersimilarity* to define highly similar DNA methylation patterns that likely arise during early embryonic stages of monozygotic twins and are unlikely explained by DNA sequence variance (Van Baak et al., 2018).

A comprehensive study that aimed directly for untangling genetics and epigenetics by coupling transcriptional variance to detailed genetics and epigenomics in blood cells pointed out that in healthy conditions, variation in *cis*-regulatory DNA elements is a more important determinant of transcriptional variance than epigenetic patterns of DNA methylation, or single histone modifications (Chen et al., 2016). Interestingly, Chen and colleagues excluded external regulators of blood cell epigenetics by selecting blood samples based on the absence of elevated inflammatory factors (‘healthy individuals’), though, deselecting for environmental variation (Chen et al., 2016). Indeed, the transcripts that did correlate more independent of DNA sequence differences to changes in epigenetic marks were regulators in pathways involved in pathogen response, including NfKB, a key regulator of aging related inflammation and linked to aging and PD (Collins et al., 2012; López-Otín et al., 2013). From a different perspective, the efficiency of reprogramming fibroblasts can be increased by inhibition of several epigenetic modulators, suggesting that specific epigenetic mechanisms that act as facilitators of differentiation and others as gate keepers of cell fate (Onder et al., 2012). In summary, epigenetic mechanisms may become of more importance when a cell is challenged by external cues or forced to change fate, as we will exemplify below.

Other ‘independent’ epigenetic phenomena that gathered much attention are epigenetic clocks, that have the potential to predict chronological age very accurately in healthy aging by measure of cumulative methylation states of small, defined subsets of CpGs (Hernandez et al., 2011; Horvath, 2013). Strikingly, these subsets show large parallels in age-related changes between different tissues and individuals. Over the years especially ‘Horvaths clock’ has been tested as a biological aging biomarker for aging related diseases including PD and Huntington (HT) that show a ‘higher epigenetic age’ while supercentenarians and their offspring have opposing profiles (Horvath and Ritz, 2015; Horvath et al., 2016). It should be noted that the epigenetic clock shows very mild changes in PD samples compared to the effect size of other age related diseases which suggest only a weak link between global DNA methylation dysregulation and PD. Furthermore, it is uncertain what drives epigenetic clocks, but low rate stochastic methylation following for instance DNA repair (Ding et al., 2016) seems to explain clocks better than a few random unrepaired mutations in *cis-*regulatory DNA elements that coincidentally occur. However, the fact that the mechanisms that underlie the (un)even running of epigenetic clocks remain elusive prevents to determine if they are of any biological relevance.

## Epigenetic Drift: Concordance in Discordance?

Opposing to epigenetic clocks is epigenetic drift, which has been defined as “the collection of methylated CpGs that are associated with age within an individual but are not common across individuals” (Jones et al., 2015). Fraga and colleagues, were among the first to study epigenetic drift in detail. In their hallmark publication, one-third of monozygotic twins had discordant methylomes, suggesting that epigenetic drift requires a trigger that may be enclosed in divergent lifestyles (Fraga et al., 2005). Even though the principle of epigenetic drift is based on an increase in discordance between individuals, an increase in concordance has also been observed in the healthy aging brain (Oh et al., 2016). Like epigenetic clocks, epigenetic drift (epigenetic discordance) may serve as a biomarker for aging as predictor of longevity, which is in line with findings that put epigenetic drift under the influence of caloric intake (Maegawa et al., 2017). Importantly, DNA methylation discordance is not equal to all types of DNA elements, as epigenetic drift may especially occur in intergenic regions compared to promoters of active genes, suggesting that epigenetic maintenance mechanisms have divergent fidelity depending on the type of chromatin or DNA element (Hindorff et al., 2009; West et al., 2013; Issa, 2014; Jones et al., 2015; Oh et al., 2016).

## DNA Methylation and the *SNCA* Gene

**FIGURE 2:**
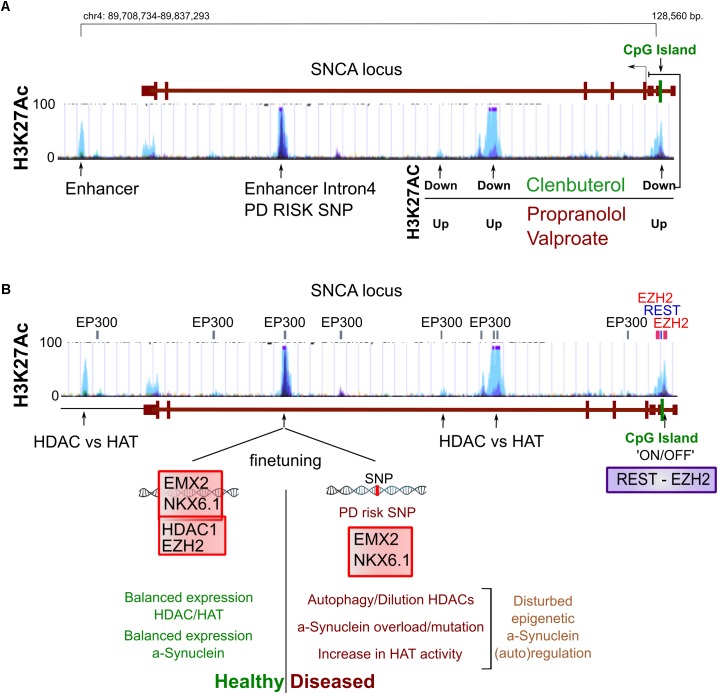
Epigenetic regulation of the *SNCA* gene. **(A)** Several sites of H3K27 acetylation are found to be regulated by β2AR agonists (Clenbuterol/Salbutamol) and reversely by antagonists but also valproic acid (VPA). From the current perspective, an enhancer in intron 4 is involved in autoregulation of *SNCA* to finetune its expression, which may be disturbed by a PD risk SNP that blocks the binding of EMX2/NKX6.1 and subsequently the recruitment of the repressing epigenetic factors HDAC1 and EZH2 **(B)**. At the proximal promoter, a CpG seems involved in silencing *SNCA* in non-expressing cells, perhaps via the regulation of REST binding and the recruitment of EZH2, which have binding sites at the same locus **(B)**. Altogether, under unchallenged, healthy conditions, with HAT and HDAC activity in balance, α-Syn may autoregulate its expression level. However, in PD conditions, autophagy or other forms of HDAC inactivity may lead as well as SNPs or mutations in regulatory elements to α-Syn overload that in turn cannot be buffered properly by autoregulation of *SNCA* levels via epigenetic mechanisms.

The question if drift of the DNA methylome is involved in PD has been approached by investigating the methylation states of the α-Syn encoding gene (*SNCA*) and its regulatory elements for the following reasons: firstly, as previously mentioned, an increase of *SNCA* alleles resulting from multiplication correlates with an increased onset of PD and severity of the disease. Secondly, detailed *in vitro* and GWAS studies have proposed *cis*-regulatory elements that are associated to PD and potentially regulated by methylation. Thirdly, α-Syn transcript levels correlate to protein levels, together suggesting that transcriptional deregulation is key in disease development of at least a subset of PD patients (Nalls et al., 2014; Soldner et al., 2016; Guhathakurta et al., 2017a). α-Syn accumulates in Lewy Bodies in a broad array of degenerative diseases (next other abundant neuronal proteins) (Shults, 2006; Armstrong et al., 2008; Goedert et al., 2013), though we have to note that in some cases of PD this may represent a lack of protein homeostasis in the absence of transcriptional changes and even lower (protein) levels have been observed in sporadic PD (Zheng et al., 2010; Quinn et al., 2012; Mariani et al., 2016). Although the regulation of the *SNCA* gene by DNA methylation is thoroughly investigated, many findings are inconclusive and sometimes contradictory, which is in line with the lack of transcriptional changes in some of the corresponding studies (Guhathakurta et al., 2017a). A commonly used approach is treatment with DNA Methyl Transferase (DNMT) inhibitors *in vitro* and data originating hereof favors DNA methylation dependent repression of *SNCA*. The best studied regulator of *SNCA* is a CpG island embedded in intron 1 (Figure 2A) that seems to have this affect presumably in cells that have low endogenous levels of α-Syn, like most cells-lines do. In PD, but also in neurons of non-PD individuals, differentiation toward DA neurons seems accompanied by hypomethylation, which is supported by strong hypomethylation of intron 1 in both experimental groups, suggesting a developmental ‘on/off’ state (de Boni et al., 2015; Basu et al., 2017; Guhathakurta et al., 2017b).

## Global DNA Methylation Changes and PD

Instead of local misprinting of epigenetic mechanisms at a single locus, global (total per cell) regulation of epigenetic marks seems intuitively related to neuronal homeostasis. In PD, global reduction of DNA methylation (∼30%) has been found to correlate to elevated levels of α-Syn and associated to sequestering of DNMT1 outside the nucleus, perhaps functioning as feed forward mechanism (Desplats et al., 2011). In comparison, a study on Alzheimer disease (AD) brains suggested a reduction in methylation of up to ∼20% (Chouliaras et al., 2013). Altogether, reports are scarce and there is not (yet) a broad consensus if global changes in DNA methylation occur in PD.

From a different perspective, global changes in DNA methylation induced by conditional genetic models or in disease have revealed hints on the potential role of global DNA methylation defects for DA neuronal maintenance. For instance, Rett syndrome is caused by an X-linked mosaic pattern of MeCP2 expression, which binds to methylated CpGs in gene bodies of active genes, leading to miscommunication between methylated DNA and other repressive epigenetic mechanisms in the maturing brain of young children (Lyst and Bird, 2015). As Rett syndrome progresses, also motor skills are being affected and DA neurons have been shown to loose functions in a cell autonomous manner (Samaco et al., 2009; Gantz et al., 2011). However, no reports of a widespread loss of neurons has been reported in Rett syndrome patients. Moreover, in mouse models and in contrast to MeCp2 overexpression, MeCp2 defects seems largely reversible (Guy et al., 2007; Taylor and Doshi, 2012). In line with this, global DNA hypomethylation has been studied in mice lacking DNMT1 and DNMT3 in mature (cortical) neurons leading to a ∼50% loss of global DNA methylation that predominantly affected neuronal functions involved in plasticity. However, no decreased lifespan or typical behavioral abnormalities have been observed in those models (Feng et al., 2010).

Part of the DNA methylome is expectedly rigid, but a relatively high turnover may occur especially on a selection of genes affected by direct environmental cues and neuronal activity instead. More specifically via hydroxymethylation through the activity of the ten eleven translocation (TET) enzymes that oxidize 5-methylcytosines (5mCs) and promote locus-specific reversal of DNA methylation (Kaas et al., 2013; Guo et al., 2014). In a likely scenario, the actively regulated genes may be MeCp2 dependently regulated under the influence of the mCpG/hCpG balance (Kinde et al., 2015). Interestingly, of the three Tet enzymes, Tet1/2 seem largely redundant while loss of Tet3 results in fatal neonatal developmental defects (Dawlaty et al., 2013; Tsukada et al., 2015). Of significance to PD, Tet3 has been proposed as regulator of autophagy and lysosomal genes in neurons (Jin et al., 2016). Next to nuclear DNA, there is increasing evidence that methylation affects the regulation of mtDNA via a specific mitochondrial mtDNMT together with TET proteins that may be increasingly active in aging neurons based on the increase in mtDNA hydroxymethylation (Dzitoyeva et al., 2012; Lardenoije et al., 2015; Tsukada et al., 2015; Delgado-Morales and Esteller, 2017). Altogether, while the current link between deregulated DNA methylation and PD neurodegeneration seems weak, the turnover of DNA methylation via hydroxymethylation has opened up as a new potential factor in regulating neuronal homeostasis.

## Histone Acetylation Balance in PD

Central in genome wide gene regulation are histone acetylation dynamics. Hereby, negatively charged acetyl groups neutralize positively charged histone lysine residues thereby weakening the interaction with the negatively charged DNA and generally resulting in gene activity (Bannister and Kouzarides, 2011). Several classes of histone acetyltransferases (HATs) and histone deacetylases (HDACs) regulate the acetylation state of dozens of histone lysine residues involved in key neuronal functions (Gräff and Tsai, 2013).

The association between histone acetylation and PD has been a subject of dynamic discussion over the past decade. Initially, various studies suggested both histone hyperacetylation following *in vitro* cellular stress by the administration of pesticides as well as protection against Lipopolysaccharides (LPS), α-Syn and 1-methyl-4-phenyl-1,2,3,6-tetrahydropyridine (MPTP) using HDAC inhibitors (van Heesbeen et al., 2013). However, most HATs and HDACs are not completely restricted to histones, especially the aging related sirtuins (Sirt) can regulate a variety of proteins including epigenetic modifiers, not only by means of deacetylation, but also by adding other acyl groups or acting completely outside of the nucleus (Martínez-Redondo and Vaquero, 2013; Bheda et al., 2016). We here discuss especially HDACs that have a profound effect on epigenetics, but some may still have (un) known additional roles in PD.

Perhaps in contrary to DNA methylation, evidence that an imbalance of histone acetylation dynamics is a key component in PD, accumulated in recent years. Several detailed studies more directly related histone hyperacetylation to human cases of PD in both a genetic and epigenetic fashion. First of all, a single nucleotide polymorphism (SNP) in intron 4 of the *SNCA* gene has been found to disturb the binding site of the repressive complex EMX2/NKX6.1 (Figure 2). The lack of binding affects its enhancer activity and resulting in disproportional expression of the SNP containing alleles as a fraction of tightly regulated total levels of *SNCA* (Soldner et al., 2016). On top of this, it is known that these enhancers are acetylated on H3K27 when active (Creyghton et al., 2010). Interestingly, EMX2 function is dependent on HDAC recruitment and activity and NKX6.1 is a known recruiter of HDAC1 and EZH2, the H3K27 methyltransferase (Chen et al., 2011; Li F. et al., 2016, Li H.-J. et al., 2016). As such, EMX2/NKX6.1 binding results in deacetylation and likely in subsequent methylation of H3K27 that would lead to an increasingly repressive chromatin state (Figure 2B).

Secondly, another study investigated the same principle from a different angle, starting their search by screening for substances that repress *SNCA* expression *in vitro* and found several β2-Adrenoreceptor (β2AR) agonists to repress *SNCA* expression (Mittal et al., 2017). One of these agonists, Salbutamol that is able to pass the blood–brain barrier and was found to associate with a reduced risk for PD. Whereas antagonists correlate to an increased PD risk. Mittal and colleagues further elegantly show that H3K27 acetylation of *SNCA* enhancing elements can be regulated in a level dependent manner by both β2AR agonists (down) and antagonists (up) (Mittal et al., 2017) and confirmed that β2AR regulation of *SNCA* is HDAC dependent using the HDAC inhibitor Valproic acid (VPA), an HDAC inhibitor that has highest affinity for class I HDACs where HDAC1 and HDAC2 belong to. On top of that, VPA has been associated with PD in the past, and withdrawal of the drugs has proven to alleviate PD symptoms raising the intriguing question if some of the PD symptoms are reversible by targeting epigenetics (Mahmoud and Tampi, 2011). In summary, broad HDAC inhibition seems to deteriorate DA neuronal function, upregulates *SNCA* expression and is associated with the function of a SNP in a crucial enhancer of *SNCA* that is a risk factor of PD.

A glance of epigenetic dysregulation of ClassI HDACs in neurodegeneration has been provided by Gräff and colleagues in a study where they primarily underscore the possibility that some of the loss of functions associated to neurodegeneration may actually be reversible via targeting acetylation (Gräff et al., 2012). However, they also point out the divergent functions that HDAC1 and HDAC2 may fulfill in neurodegenerative conditions. In line with this, REST, a broad neuronal regulator of plasticity and recruiter of HDAC1/2 and Tet3 have also been found protective in models of neurodegeneration (Hwang et al., 2017). We have to note that in different forms of degeneration, the balance of histone acetylation and deacetylation may be deregulated via independent routes or even opposite. We can exemplify this by comparing PD and Huntington disease (HD). For PD and aging (global) hyperacetylation has been suggested as a consequence of autophagy of HDACs or upregulation of HAT activity, affecting the actual enzymatic balance in favor of hyperacetylation (Song et al., 2010, 2011; Park et al., 2016). Opposite, mutations in the HD factor Huntingtin (Htt) have been suggested to disturb cytosolic binding of REST1 to the protein, leading to increased HDAC recruitment to responsive genes resulting in a decreased acetylation state of REST1 targets (Hwang et al., 2017).

Altogether, in recent years there has been an increasing number of studies supporting the importance of global H3K27 acetylation state in PD, in particular via the regulation of PD associated α-Syn and via a reduction in HDAC activity, or a lack of their recruitment (Table 1).

**Table 1:** Age and PD related mechanisms linked to epigenetics.

Effector mechanism	Epigenetic component	Mode of action/description	Reference
DNA repair Oxidative stress	Sirt1 recruitement	Age/DNA damage related genomic redistribution of Sirt1 to assist DNA repair and promote genomic stability may influence Sirt1 gene-regulatory functions elsewhere	Oberdoerffer et al., 2008
DNA repair Oxidative stress	DNMT recruitement, DNA methylation	Oxidative stress leading to DNA mutation precedes the recruitment of DNMT1 by DNA repair mechanisms	Mortusewicz et al., 2005
DNA repair MMR	DNMT/Sirt1 recruitement	Mismatch repair recruits DNMT1 and Sirt1, authors hypothesize epigenetic silencing of repair sites to avoid interference with transcriptional machinery	Ding et al., 2016
DNA repair NHEJ	H3K36 methylation	Fumarate induced downregulation of KDM2B activity may lead to increased H3K36me2 to promote repair of DSBs	Jiang et al., 2015
DNA repair NHEJ	Histone degradation	DNA repair and general histone degradation are coupled in yeast. Recombination rate increase following increased general histone degradation	Hauer et al., 2017
Aging	H3.3 levels	H3.3 levels are a longevity factor in nematodes. H3.3 is the only factor newly incorporated H3 histone variant in post-mitotic neurons	Adam et al., 2013; Piazzesi et al., 2016
DNA repair	Chromatin density	Higher mutation rate observed in open chromatin of human neurons by single neuron genomics	Lodato et al., 2015
Autophagy/DNA repair	5hmC/Tet3	The DNA methylation oxidase Tet3 is specifically targeted to lysosomal and base excision repair genes and potentially protects against neurodegeneration	Jin et al., 2016
MtDNA regulation	5hmC/Tet proteins	DNA methylation affects the regulation of mtDNA via a specific mitochondrial mtDNMT together with TET proteins that may be increasingly active in aging neurons considering the age related increase in mtDNA hydroxymethylation	Dzitoyeva et al., 2012; Lardenoije et al., 2015; Tsukada et al., 2015; Delgado-Morales and Esteller, 2017
Autophagy	Histone hyperacetylation Decrease HDACs	Autophagy of HDACs may induce global hyperacetylation in PD	Park et al., 2016
Pesticides	Histone hyperacetylation Increase HATs	Pesticides may induce global hyperacetylation following the HAT P300 induction	Song et al., 2010, 2011
Gene deletion, Neurodegeneration	H3k27me3 EZH1/2	Neuronal depletion of H3K27me3 leads to neurodegeneration *in vivo* of matured neurons	von Schimmelmann et al., 2016
PD, epigenetic clock Horvaths clock	DNA methylation	*Mild* acceleration of Horvath’s epigenetic aging clock in blood cells of PD patients	Horvath and Ritz, 2015
PD, protein sequestering	DNMT1	Global reduction of DNA methylation and sequestering of DNMT1 outside the nucleus in PD	Desplats et al., 2011


More speculative, two studies have reported an antagonistic role for the wild-type α-Syn protein against p300 acetylating activity (Kontopoulos et al., 2006; Jin et al., 2011), which could imply a feedback mechanism to limit not only global acetylation, but perhaps also reducing acetylation of it’s own enhancer (Figure 2B). From a disease perspective, MPTP administration may as well lead to global hyperacetylation at H3K27 and a variety of other lysine residues, among which most prominently H3K9. These results have been partly based on PD post-mortem material in a study that again puts downregulation of class I HDACs—via autophagy—forward as cause (Park et al., 2016). It will be interesting to see whether the PD risk SNP in the intron 4 enhancer of *SNCA* will become more important in stressed situations or changed activity of epigenetic modifiers, when it seems likely that more is demanded of gene regulatory feedback mechanisms (Figure 2B).

Finally, the epigenetic state that is opposite to H3K27 acetylation, is H3K27 methylation, which me3 state marks poised enhancers instead of active (Zentner et al., 2011). While deletion of several DNMTs seems relatively harmless to post-mitotic neurons (although only ∼50% of global methylation is lost) as we have described earlier, loss of the H3K27 methyltransferases Ezh1/2 leads to neurodegeneration in both D1 and D2 DA receptor-positive neurons, while loss of Ezh2 leads to derepression of neurodegeneration associated genes (von Schimmelmann et al., 2016). Opposite, hypermethylation of H3K27 is also associated with neurodegeneration of the cerebellum in ataxia-telangiectasia (Li et al., 2013). Altogether, several lines of evidence point toward a central role of histone regulation via H3K27 that may be deregulated globally via increased acetylation, having harmful effects through dysregulation of *SNCA* and potentially other genes.

## Neuroinflammation and Epigenetics

A role for inflammatory pathways in neuronal maintenance and PD has been clearly established (Table 1). Recently, neuronal Interferon-β regulation has been proven vital in maintenance of dopaminergic neurons and to avoid Lewy Body formation and neurodegeneration (Ejlerskov et al., 2015). Much of the additional evidence has emerged around broad inflammatory regulation by NfKB signaling, in particular the p65/RelA subunit, that translocates to the nucleus of DA neurons in neurodegenerative conditions while inhibition is neuroprotective. Furthermore, intraperitoneal LPS administration leads to DA neurodegeneration, suggesting that non-neuronal or peripheral inflammation pathways may as well be a local burden for DA neurons (Hunot et al., 1997; Ghosh et al., 2007;Qin et al., 2007; Collins et al., 2012).

**FIGURE 3:**
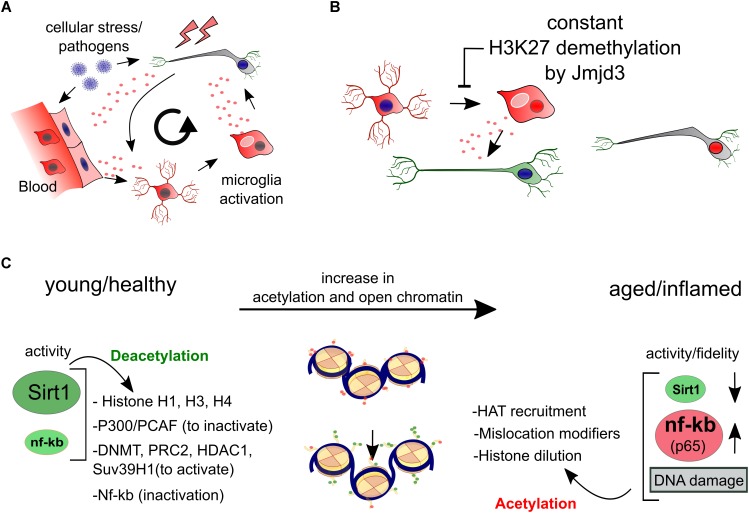
Epigenetics of inflammation. **(A)** We have schematized the interaction between cellular stress like oxidation levels, local and global pathogen responses and inflammation that all have the potential to activate microglia which in turn can be harmful for surrounding (healthy) neurons. **(B)** The activation of both microglia as well as macrophages can be seen as a developmental process of differentiation that is initiated by the sole downregulation of Jmjd3, a H3K27 demethylase, even in the absence of other stimuli. **(C)** In aging and inflammation, a reduction of the activity and expression of Sirt1 has been observed, while inflammatory factors like NfKB (p65) are generally activated. Sirt1 deacetylates a large portion of histone residues at several subunits. Next to this, activation of several repressive epigenetic enzymes by deacetylation can lead to a further global silencing of chromatin. Opposite, NfKB has been linked to regulation of aging and longevity regulated epigenetic factors, favoring accelerated aging. Combined and with the involvement to others, a Sirt1/NfKB balance shift toward a more inflammatory state in aging and perhaps PD seems to increase a global increase in euchromatin.

It is also widely appreciated that accumulating reactive microglia surrounding neurons in the PD brain can become harmful following their prolonged secretion of interleukines and TNF-α (Hanisch, 2002; Doorn et al., 2012). Interestingly, microglia can be activated—alike macrophages—simply by reduction of the H3K27me3 demethylase Jmjd3 (De Santa et al., 2009). It seems that genes that maintain microglia inactivated need to be silenced by a switch of bivalent (H3K4me3/H3K27me3) loci toward repressed loci (De Santa et al., 2009; Tang et al., 2014) (Figure 3A,B).

Epigenetics of inflammation show more remarkably independent aspects. In a comprehensive study, genes involved in inflammatory pathways like NfKB were found epigenetically regulated, relatively unrelated to their genetic background when compared to other blood cell transcriptional networks (Chen et al., 2016). This seems intuitive since these pathways have to facilitate the defense against external pathogens, responding predominantly to environmental cues while preserving parts of their cell identity. This raises the question if prolonged exposure of neurons to inflammatory cues may in the end deregulate unwanted transcriptional pathways by ways of epigenetic drift.

Possible candidates for inflammatory related epigenetic drift is Sirt1, a class III HDAC, that acts via broad deacetylation of the NfKB gene, various histone residues, transcription factors and epigenetic enzymes.

Although Sirt1 can have other functions, the majority of its roles are likely via broad epigenetic silencing that may happen directly, through histone deacetylation of H1, H3, H4, or indirectly via the activation of epigenetic silencers like DNMTs, Polycomb Repressive Complex 2 (H3K27Me3), HDAC1 and Suv39H1 (H3K9me3), or the silencing of the HATs: P300/PCAF (extensive review, Jing and Lin, 2015). Overall it has been suggested that Sirt1 inactivation may follow upon oxidative stress that causes a lack of NAD+, the substrate for Sirtuin activity. Inactivity can lead to failure of NfKB repression by Sirt1 and there is some evidence that that may occurs in PD (Braidy et al., 2011; Singh et al., 2017). This would again favor the idea of increased global acetylation in PD (Figure 3C).

Regulation by NfKB itself may also effect epigenetic regulation directly, and opposite to Sirt1 may particularly induce histone acetylation in neurons by recruiting EP300 to chromatin, which is specifically facilitated by nuclear transactivation of RelA/p65 (Oeckinghaus and Ghosh, 2009; Federman et al., 2013).

In other non-neuronal models of cancer, NfKB has been linked to inhibition of nuclear receptor binding SET domain protein 1 (NSD1) and SET domain containing 2 (SetD2), two H3K36 histone methyltransferases, which are linked to longevity, though not general to chromatin silencing (Sen et al., 2015) (Figure 3C). Altogether, from the initial cue to global neuronal transcriptional changes, inflammation pathways have a broad potential to act independently through epigenetic changes in blood cells, microglia and neurons by increasing global histone acetylation. However, to which extend these pathways are cell autonomously or environmentally induced in neurons, and if such would lead to changes in epigenetic neuronal dynamics is largely open.

## DNA Repair and Epigenetic Fidelity

Genetics and Epigenetics are evidently entangled when it comes to cellular stress responses. In PD, DA metabolism, dysfunction of autophagy, mitochondrial respiration and inflammation have all been suggested to potentially elevate levels of oxidants (Dias et al., 2013; Blesa et al., 2015). One of the consequences of extensive oxidation is damage to DNA and proteins that need to be repaired. Inefficient repair of DNA via the nucleotide excision repair (NER) machinery that acts on active genes, can cause accelerated aging and a PD phenotype (Sepe et al., 2016). Interestingly, single nucleotide mutations positively correlate to open chromatin and exons (e.g., DNAI/H3K4me3) in neurons, which is opposite to dividing cancerous cells (Lodato et al., 2015; Tubbs and Nussenzweig, 2017). It seems that densely packed chromatin is less affected by mutations in differentiated neurons, or that the repair mechanisms are less effective in open chromatin. It is known that Sirt1 and suppressor of variegation 3–9 homolog 1 (Suv39H1) form facultative heterochromatin to protect cells from oxidative stress in cancerous cells (Bosch-Presegué et al., 2011). We theorize that fully differentiated neurons have accumulated more protective heterochromatin than cancerous cells during their maturation. In addition, Sirt1 affects transcription following relocation to facilitate genome stability of double strand breaks (Oberdoerffer et al., 2008). Since Sirt1 and its co-factor NAD+ are expected to decline with age and in PD, this may add up to less protection against oxidative stress combined with further dilution and a lack of fidelity of Sirt1 epigenetic and non-epigenetic regulation (Oberdoerffer et al., 2008; Imai and Guarente, 2014; Jing and Lin, 2015).

Additional mechanisms that may well be conserved to neuronal aging is the localization of fumarate to DNA damage sites to facilitate non-homologous end joining (NHEJ) DNA repair and downregulates the H3K36me2 demethylase, KDM2B, leading to increasing H3K36me2 (Jiang et al., 2015). Perhaps, such elongation marks are coupled to histone degradation, which by facilitates DNA repair (Hauer et al., 2017). Interestingly, a global decrease of histone density is a conserved hallmark of aging (Pal and Tyler, 2016). Herein, the post-mitotically incorporated histone H3.3 seems a key factor and is recently found as pro-longevity factor in nematodes (Adam et al., 2013; Piazzesi et al., 2016). Since H3.3 is the main H3 variant that is incorporated in post-mitotic cells, H3.3 expression may be essential in maintenance of nucleosome density, proper DNA repair and post-repair gene regulation (Adam et al., 2013). Appropriate global levels of nucleosomes seem necessary for instance since they are precisely placed directly downstream intron–exon boundary sequences (Andersson et al., 2009), as such, changes in density of nucleosomes could be another mechanism that influences epigenetic fidelity in aging or diseased neurons.

## Conclusion

In recent years there has been an increasing number of studies supporting the importance of global H3K27 acetylation in PD, in particular via deregulation of *SNCA*, possibly caused by a reduction in HDAC activity, or a lack of its recruitment. In diseases associated to PD, findings support a comparable role for Sirt1. Pathways that induce hyperacetylation or epigenetic infidelity include neuroinflammation and endogenous cellular stress, like deregulation of autophagy, oxidation and DNA repair mechanisms (listed in Table 1). Overall, during the last decades, opposite to an increased appreciation of the DNA sequence as map for the transcriptome in healthy cells, the latest insights point toward a more crucial role for epigenetics when gene-regulatory buffers are put to the test which may be the case in a state of PD. Importantly, if such global changes to histone epigenetics will be repetitively found in human dopamine neurons at early-PD stages or in aging human DA neurons, their corresponding histone modifiers will be ideal targets for small molecule inhibition therapeutics in the future.

## Author Contributions

All authors listed have made a substantial, direct and intellectual contribution to the work, and approved it for publication.

## Conflict of Interest Statement

The authors declare that the research was conducted in the absence of any commercial or financial relationships that could be construed as a potential conflict of interest.
